# Water Content Differences Have Stronger Effects than Plant Functional Groups on Soil Bacteria in a Steppe Ecosystem

**DOI:** 10.1371/journal.pone.0115798

**Published:** 2014-12-29

**Authors:** Ximei Zhang, Albert Barberán, Xunzhi Zhu, Guangming Zhang, Xingguo Han

**Affiliations:** 1 State Key Laboratory of Forest and Soil Ecology, Institute of Applied Ecology, Chinese Academy of Sciences, Shenyang 110016, China; 2 Cooperative Institute for Research in Environmental Sciences, University of Colorado, Boulder, Colorado, United States of America; 3 School of Biology and Chemical Engineering, Jiangsu University of Science and Technology, Zhenjiang, Jiangsu 212018, China; 4 State Key Laboratory of Vegetation and Environmental Change, Institute of Botany, Chinese Academy of Sciences, Beijing 100093, China; Argonne National Laboratory, United States of America

## Abstract

Many investigations across natural and artificial plant diversity gradients have reported that both soil physicochemical factors and plant community composition affect soil microbial communities. To test the effect of plant diversity loss on soil bacterial communities, we conducted a five-year plant functional group removal experiment in a steppe ecosystem in Inner Mongolia (China). We found that the number and composition type of plant functional groups had no effect on bacterial diversity and community composition, or on the relative abundance of major taxa. In contrast, bacterial community patterns were significantly structured by soil water content differences among plots. Our results support researches that suggest that water availability is the key factor structuring soil bacterial communities in this semi-arid ecosystem.

## Introduction

Human activities are accelerating the loss rate of plant and animal diversity [1], [2]. Consequently, a large body of manipulative experiments [3], [4], field investigations [5], [6] and theoretical analyses [7], [8] have been conducted to identify the influence of biodiversity loss on ecosystem function, processes and services, and most of these studies suggest that a large pool of species is needed to sustain ecosystem processes in changing environments [9]. Although many functions have been examined, attention has primarily focused on those directly associated with higher organisms such as plant productivity [10].

Plants provide a source of organic carbon to belowground microorganisms, while microbes decompose and make nutrients available to plants [11]. This relationship presumes that the plant community might be an important factor structuring the belowground microbial community [12], [13]. For example, plant diversity has been shown to influence both soil microbial activity [14] and soil microbial growth [15]. Additionally, invasive plant species have been found to change the composition of associated belowground microbial communities [16].

In this study, we investigated the influence of plant functional diversity loss on soil bacterial communities in a homogeneous steppe ecosystem in Inner Mongolia (China) by the removal of naturally present plant functional groups (PFG). Our main objective was to determine the effect of PFG removal on soil bacterial diversity and community similarity, as well as on the relative abundance of major taxonomic groups. As many investigations have reported that plant communities affected soil microorganisms [12], [17], our initial hypotheses were as follows: (1) soil bacterial diversity should increase with the number of PFG; (2) different PFG combinations should have different effects on soil bacterial diversity; (3) PFG number and different PFG combinations should be reflected by soil bacterial community patterns; and (4) PFG number and different PFG combinations should have different effects on the relative abundance of major bacterial taxa (phyla/classes).

## Materials and Methods

### Study site and experimental design

This study is part of the Inner Mongolia Grassland Removal Experiment (IMGRE) from the Chinese Academy of Sciences. Our field studies did not involve endangered or protected species, so no specific permissions were required for the location/activity. The experiment was conducted in a typical steppe semi-arid ecosystem (43°38′N, 116°42′E). The mean annual temperature is ∼0.3°C and the average precipitation is 346 mm per year mostly occurring from July to September. The soil is dark chestnut with sandy and silty loam in texture and corresponds to a Calcis-orthic Aridisol according to US Soil Taxonomy [18]. The vegetation is dominated by *Leymus chinensis*, *Agropyron michnoi*, *Achnatherum sibiricum*, *Cleistogenes squarrosa* and *Stipa grandis*.

All plant species in this ecosystem were classified into five PFG based on their life forms [19], [20]. Among these PFG, perennial rhizome grass (PR), perennial bunchgrasses (PB) and perennial forbs (PF) comprised >99% of the total aboveground biomass (Table 1). Therefore, we only investigated the effects of these three PFG and their combinations. A full combinatorial design was employed with a total of eight (2^3^) treatments (Table 2) and five replicates (in five random blocks) for each treatment. PFG diversity gradients were established in early July every year from 2005 to 2009 by manual removal of the aboveground biomass of non-target plants in each plot. Stems and leaves were removed by clipping at the surface while taking great care to reduce disturbance to soil and other plants [19], and the clipped plant material was removed from the plots. Previous studies at IMGRE using the same experimental setup have analyzed the response of soil carbon and nitrogen pools [20] and the response of nitrogen-cycling genes [21].

**Table 1:** Plant functional groups and their properties.

Plant functional groups	Species number of this PFG	Representative species	Leaf δ^13^C value (‰)^a^	C:N (atomic ratio)	Root/shoot ratio	Aboveground biomass (g.m^-2^)
Perennial rhizome	1	*Leymus chinensis*	−26.13	28.99	4.0	102.10
Perennial bunchgrass	7	*Agropyron cristatum; Cleistogenes squarrosa; Stipa grandis*	−22.40	33.53	2.8	139.88
Perennial forbs	>20	*Allium bidentatum; Carex korshinskyi; Potentilla bifurca*	−26.81	22.89	3.1	40.43

a Leaf δ^13^C values are related to plant water use efficiency.

**Table 2:** Experiment design and soil physicochemical characteristics under different plant functional groups (PFG) treatments.

Treatments	PFG number	PB	PR	PF	TC (g/kg)	TN (g/kg)	NH_4_^+^-N (mg/kg)	NO_3_^−^-N (mg/kg)	Water (%)	pH
1 (control)	3	+	+	+	17.11±1.48	1.82±0.19	16.32±2.96	1.55±0.45	4.49±0.52	7.19±0.11
2	2	+	+	−	17.61±1.48	1.90±0.15	16.05±3.88	1.54±0.25	4.77±0.51	7.18±0.11
3	2	+	−	+	22.44±1.33	2.28±0.16	10.55±1.40	1.66±0.15	4.68±0.0.30	7.21±0.12
4	2	−	+	+	19.46±1.32	1.71±0.30	11.26±1.58	1.07±0.19	4.29±0.60	7.22±0.08
5	1	+	−	−	19.07±1.47	1.99±0.14	15.64±3.25	2.02±0.59	4.17±0.60	7.19±0.09
6	1	−	+	−	17.38±2.24	1.91±0.22	17.87±4.17	1.42±0.09	4.44±0.75	7.20±0.10
7	1	−	−	+	20.52±0.66	2.20±0.08	12.90±2.05	3.96±1.01	4.77±0.54	7.23±0.10
8	0	−	−	−	19.38±1.85	2.07±0.19	9.87±1.53	5.73±1.66	4.16±0.35	7.19±0.11
Effect of PFG number (ANOVA)				*F*	0.313	1.122	0.653	15.145	0.331	0.0023
				*P*	0.579	0.296	0.424	<0.001	0.568	0.962

“−” and “+” represents the corresponding PFG as being absent (removed) or present (not removed), respectively. The values represent mean ±se.

### Sampling, soil characteristics, molecular analyses and 16S rRNA analyses

Soil samples were taken on 22^th^ June 2010. Because the plant roots were generally of >10 cm depth [20], four soil cores (10 cm deep, 3.5 cm diameter) were collected from each plot at random and thoroughly mixed, part of which was used to measure soil physicochemical indices and the rest was frozen for DNA extraction. Soil characteristics were measured as described before [21] (see Table 2 for a summary under the different PFG treatments).

Molecular and 16S rRNA sequence analyses followed the procedures described previously [22]. Briefly, we extracted DNA from 0.5 g of mixed soil using the Fast DNA SPIN kit for soil according to the manufacturer's instructions (Qbiogene, Carlsbad, CA, USA). The primers 27F and 338R, which were found to behave well in community-level pyrosequencing based analysis [23], [24], were used to amplify the V1 and V2 hyper-variable regions of the 16S rRNA gene. Equal molar concentrations of PCR products for each sample were then pooled and sequenced in a Roche 454 Genome Sequencer FLX Titanium system at Shanghai Majorbio Bio-pharm Technology Co., Ltd. The 16S rRNA reads were analyzed with the Mothur software (Version 1.19) [25]. Reads shorter than 150 nucleotides or with ambiguous characters were removed. Putative chimeric sequences were also excluded using the UCHIME algorithm with default parameters [26]. As raw read counts can vary by orders of magnitude, we randomly selected 3,478 reads for each sample. All these sequences (3,478×40) were clustered into operational taxonomic units (OTUs) using the consensus 97% identity threshold.

Sequence reads have been deposited in the National Center for Biotechnology Information Sequence Reads Archive (accession no. SRA057669). Under this accession number, not only the DNA sequences from this study are deposited but also sequences of 66 samples from three other experiments conducted at the same site [22], [27], [28].

### Statistical analyses

Linear regressions were used to establish the relationship between the PFG number and bacterial OTU number, as well as the relative abundance of taxonomic groups (i.e., *Acidobacteria*, *Actinobacteria*, *Bacteroidetes*, *Chloroflexi*, *Firmicutes*, *Gemmatimonadetes*, *Nitrospirae*, *Planctomycetes*, *Alphaproteobacteria*, *Betaproteobacteria*, *Deltaproteobacteria*, *Gammaproteobacteria*, and *Verrucomicrobia*). Three-way ANOVAs were used to determine the effect of PFG combinations (the presence or absence of each of the three PFG) on bacterial OTU number and the relative abundance of taxa. We used the False Discovery Rate (FDR) to correct for multiple comparisons [29]. Besides OTU number and the relative abundance of different taxa, the changes in the number of PFG and PFG combinations might also affect bacterial community similarity. Community similarity was represented by non-metric multidimensional scaling (NMDS) using the Bray-Curtis distance metric after Hellinger standardization [30]. Permutational multivariate analyses of variance (PERMANOVA) after 1,000 permutations were used to determine the effect of PFG number and composition on soil bacterial community similarity [31]. All statistical analyses were carried out in R [32] with the vegan package [33].

### Assessing the potential influence of undersampling

To account for the potential influence of undersampling, we adopted two different methods. First, sequences were clustered into OTUs at the 97% threshold as well as 95% and 90% thresholds, analogous to comparing different taxonomic resolutions (i.e. comparing species, genus and order) [34], [35]. Four different alpha diversity indices (observed OTU number, Chao1, Shannon and Simpson) were calculated for each of the three OTU thresholds. Second, OTU-independent indices of phylogenetic diversity and community similarity were calculated [36], [37].

## Results

**Figure 1:**
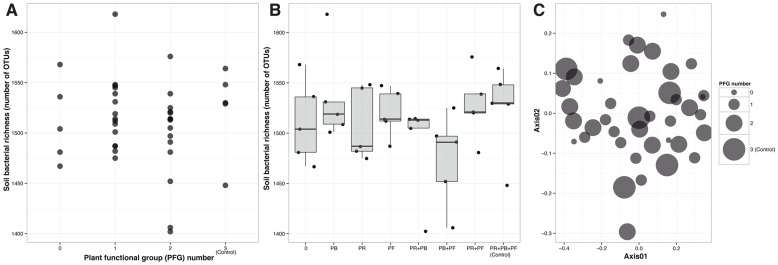
Effect of plant functional groups (PFG) on soil bacterial communities. (A) Relationship between soil bacterial OTU number and PFG number. (B) Relationship between soil bacterial OTU number and PFG combinations. (C) Non-metric multidimensional scaling (NMDS) ordination plot of the soil bacterial communities with PFG number as point size (Stress  = 0.17).

For the six soil physicochemical indices (total C, total N, NH_4_^+^, NO_3_^−^, water content and pH), as the number of PFG removed increased, only NO_3_^−^ content showed an increasing trend (Table 2). Contrary to our initial hypotheses (1) and (2), we found no effect of PFG number (Linear regression, P>0.05, Fig. 1A) or PFG combinations (Three-way ANOVA, P>0.05, Fig. 1B) on bacterial OTU number. The non-significant effects were found to be consistent across different indices of alpha diversity (with only three exceptions; Table 3). Also opposite to our initial hypothesis (3), PFG number or different PFG combinations did not have a significant effect (PERMANOVA, P>0.05) on bacterial community similarity (Fig. 1C). The non-significant effects were also consistent across different indices of community similarity (Table 4). Contrary again to our initial hypothesis (4), none of the soil bacterial taxa showed a significant effect of PFG number or PFG combinations on their relative abundances (P≥0.05 after FDR correction, Table 5).

**Table 3:** Effect of plant functional group (PFG) number and PFG combinations on different alpha diversity indices of soil bacterial community.

						PFG composition			
Diversity indexes		PFG number	PB	PR	PF	PB*PR	PB*PF	PR*PF	PB*PR*PF
97%-OTU number	*F*	0.027	1.862	1.159	0.343	0.591	0.983	3.394	1.554
	*P*	0.871	0.182	0.290	0.562	0.448	0.329	0.075	0.222
97%-Chao1	*F*	0.933	0.403	0.362	2.631	1.323	0.416	0.758	0.242
	*P*	0.340	0.530	0.552	0.115	0.259	0.524	0.391	0.626
97%-Shannon	*F*	0.011	0.960	0.581	0.159	0.296	2.004	1.435	1.614
	*P*	0.918	0.334	0.451	0.692	0.590	0.167	0.240	0.213
97%-Simpson	*F*	0.030	0.330	0.000	0.082	0.082	1.320	0.000	0.742
	*P*	0.863	0.570	1.000	0.776	0.776	0.259	1.000	0.395
95%-OTU number	*F*	0.001	2.679	0.984	0.477	0.467	0.956	5.799	2.368
	*P*	0.981	0.111	0.329	0.495	0.499	0.335	0.022*	0.134
95%-Chao1	*F*	0.954	1.041	0.702	2.154	0.205	0.125	0.851	0.004
	*P*	0.335	0.315	0.408	0.152	0.654	0.726	0.363	0.952
95%-Shannon	*F*	0.086	0.775	0.904	0.210	0.279	1.886	2.084	2.977
	*P*	0.771	0.385	0.349	0.650	0.601	0.179	0.159	0.094
95%-Simpson	*F*	0.081	0.230	0.452	0.452	0.009	2.074	0.083	0.747
	*P*	0.777	0.634	0.506	0.506	0.924	0.160	0.775	0.394
90%-OTU number	*F*	0.047	3.169	0.298	0.644	1.042	1.161	8.086	3.807
	*P*	0.829	0.085	0.589	0.428	0.315	0.289	0.008*	0.060
90%-Chao1	*F*	0.800	1.506	0.007	0.162	0.206	2.679	1.785	0.149
	*P*	0.377	0.229	0.932	0.690	0.653	0.111	0.191	0.702
90%-Shannon	*F*	0.018	2.472	0.226	0.707	0.483	1.638	2.707	6.739
	*P*	0.895	0.126	0.638	0.407	0.492	0.210	0.110	0.014*
90%-Simpson	*F*	0.273	1.384	0.195	0.022	0.022	0.541	0.086	2.162
	*P*	0.605	0.248	0.662	0.884	0.884	0.468	0.771	0.151
Phylogenetic diversity	*F*	0.012	0.091	0.194	0.099	0.002	0.000	0.063	0.001
	*P*	0.913	0.765	0.662	0.755	0.965	0.988	0.804	0.972

*refer to the significant response (*P*<0.05).

**Table 4:** Effect of plant functional group (PFG) number and PFG combinations on bacterial community similarity.

						PFG combinations			
Indices		PFG number	PB	PR	PF	PB*PR	PB*PF	PR*PF	PB*PR*PF
97%-OTU Bray-Curtis distance	*R* ^2^	0.019	0.016	0.019	0.015	0.017	0.017	0.017	0.027
	*P*	0.733	0.975	0.793	0.998	0.917	0.937	0.934	0.354
95%-OTU Bray-Curtis distance	*R* ^2^	0.021	0.018	0.020	0.014	0.018	0.016	0.016	0.028
	*P*	0.593	0.871	0.686	0.999	0.866	0.954	0.952	0.349
90%-OTU Bray-Curtis distance	*R* ^2^	0.021	0.018	0.021	0.015	0.017	0.017	0.019	0.022
	*P*	0.595	0.821	0.645	0.973	0.883	0.906	0.736	0.612
Weighted Unifrac distance	*R* ^2^	0.023	0.015	0.013	0.026	0.016	0.019	0.040	0.021
	*P*	0.475	0.819	0.872	0.428	0.764	0.639	0.166	0.564

**Table 5:** Effects of plant functional group (PFG) number and PFG combinations on the relative abundance of soil bacterial taxa.

Taxa	PFG number								PFG combination							
			PB		PR		PF		PB*PR		PB*PF		PR*PF		PB*PR*PF	
	P	P_FDR_ a	P	P_FDR_	P	P_FDR_	P	P_FDR_	P	P_FDR_	P	P_FDR_	P	P_FDR_	P	P_FDR_
*Acidobacteria*	0.671	0.893	0.756	0.856	0.968	0.968	0.721	0.876	0.513	0.943	0.113	0.685	0.763	0.827	0.979	0.979
*Actinobacteria*	0.754	0.893	0.790	0.856	0.651	0.935	0.219	0.651	0.415	0.943	0.361	0.685	0.894	0.894	0.808	0.979
*Bacteroidetes*	0.097	0.632	0.256	0.810	0.188	0.790	0.742	0.876	0.280	0.909	0.440	0.716	0.449	0.729	0.626	0.979
*Chloroflexi*	0.710	0.893	0.990	0.990	0.952	0.968	0.433	0.703	0.192	0.832	0.136	0.685	**0.004**	0.050	0.433	0.979
*Firmicutes*	0.756	0.893	0.312	0.810	0.540	0.935	**0.033**	0.434	0.598	0.943	0.369	0.685	0.102	0.316	0.450	0.979
*Gemmatimonadetes*	**0.037**	0.486	0.131	0.810	0.243	0.790	0.250	0.651	**0.046**	0.382	0.562	0.808	**0.046**	0.298	0.467	0.979
*Nitrospirae*	0.977	0.977	0.650	0.856	0.696	0.935	0.375	0.703	0.606	0.943	0.673	0.808	0.628	0.742	0.839	0.979
*Planctomycetes*	0.935	0.977	0.692	0.856	0.719	0.935	0.857	0.876	0.059	0.382	0.334	0.685	0.137	0.316	0.943	0.979
*Alphaproteobacteria*	0.633	0.893	0.790	0.856	0.434	0.935	0.193	0.651	0.968	0.968	0.711	0.808	0.593	0.742	0.383	0.979
*Betaproteobacteria*	0.293	0.893	0.756	0.856	0.549	0.935	0.148	0.651	0.798	0.943	0.746	0.808	0.259	0.481	0.896	0.979
*Deltaproteobacteria*	0.585	0.893	0.202	0.810	0.837	0.968	0.876	0.876	0.779	0.943	0.362	0.685	0.146	0.316	0.837	0.979
*Gammaproteobacteria*	0.370	0.893	0.279	0.810	0.173	0.790	0.382	0.703	0.958	0.968	0.936	0.936	0.069	0.301	0.268	0.979
*Verrucomicrobia*	0.321	0.893	0.609	0.856	0.058	0.756	0.758	0.876	0.758	0.943	0.188	0.685	0.609	0.742	0.264	0.979

a P_FDR_ refer to corrected P-values using the False Discovery Rate (FDR) method [29].

**Figure 2:**
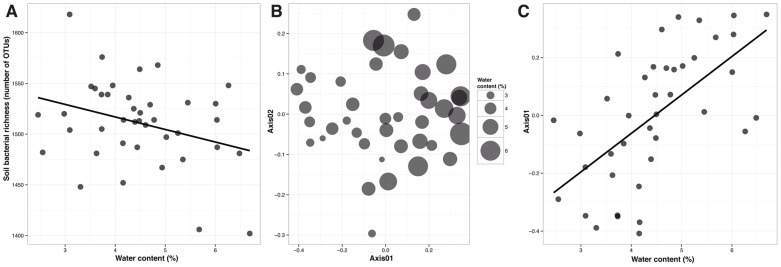
Effect of water content on soil bacterial communities. (A) Relationship between soil bacterial OTU number and water content. (B) Non-metric multidimensional scaling (NMDS) ordination plot of the soil bacterial communities with water content as point size (Stress  = 0.17). (C) Relationship between the first axis of the NMDS ordination and water content.

Given the homogeneity of soil physicochemical indices across the experiment site (Table 2), we unexpectedly detected a significant signal of the belowground soil environment on bacterial communities. Among the six soil characteristics analyzed, only water content had significant effects on bacterial communities. In particular, bacterial OTU number tended to decrease as water content increased (Linear regression, F = 4.13, P = 0.049, Fig. 2A) and bacterial community composition showed a significant effect of water content (PERMANOVA, P<0.001, Fig. 2B; Linear regression with the first axis of the ordination, F = 23.40, P<0.001, Fig. 2C). Both effects had low explanatory power: R^2^ = 0.10 and R^2^ = 0.05 for the linear regression with OTU number and for PERMANOVA with community similarity, respectively.

## Discussion

A majority of previous studies have identified a significant effect of plant communities on belowground soil microbial communities [12], [17], [38], [39]. However, this effect tended to be more prevalent under extreme (such as the removal of all plant species) or long-term treatments. Generally, a change in organic carbon resource, nutrients or other soil characteristics has been identified as the mechanisms driving this effect [38], [39]. This body of work suggests that plant identity can be a key driver of soil properties, subsequently affecting the composition and activity of soil microbial communities. Contrary to the previous research and our initial set of hypotheses, our results suggested that there was no major effect of plant functional groups on the composition and diversity of soil bacterial communities in the Chinese steppe ecosystem studied (Fig. 1). Although microbial communities have often been found to be sensitive to disturbance [40], the relationship between plants and belowground microbes might not be as close as commonly expected [41]–[44] and thus, soil microbial communities seem to be resistant to the loss of plant functional diversity in our experimental setup.

Notwithstanding, some methodological issues might be raised to explain the absence of a significant effect of PFG removal on soil microbial community composition and diversity. Although we clipped the aboveground plant biomass at the early growth season every year from 2005 to 2009, the belowground root of these perennial plants may need more time to decompose. Additionally, we classified dozens of plant species into only three functional groups in order to generate unambiguous experimental treatments. Longer treatments and/or experimental manipulations of the species diversity rather than of the number of functional groups could have enhanced the observation of significant effects.

In addition, the non-significant effect might be due to that 3,478 sequences of 16S rRNA gene could not fully represent the diversity and composition of the complex soil bacterial community. However, the non-significant influence of the experimental treatment was consistent across different analysis methods (Tables 3 and 4), demonstrating the effectiveness of the result. Actually, we have adopted the same method (focusing on 3,478 sequences of 16S rRNA gene from 454 pyrosequencing and using the statistical method of PERMANOVA) to investigate the influences of many other types of anthropogenic environmental changes on soil bacterial community in this steppe ecosystem, and have found the significant influence of some treatments (e.g. increased precipitation and nitrogen deposition) but non-significant influence of other treatments (e.g. mowing) [22], [27], [28]. It is worth emphasizing that the mowing treatment in the previous studies is very similar in nature to the PFG removal treatment in this study, and their effect was always non-significant. Taken together, our method was effective and PFG removal had very small influence on soil bacterial community.

Another plausible explanation of the non-significant effect of plant functional diversity on soil microbial communities in our experimental setup might be intrinsic to the steppe ecosystem. Most investigations that reported a significant effect were performed in relatively moist ecosystems, while our study was carried out in a semi-arid ecosystem. Our analyses suggested that even minimal differences in water content (Table 2) could have a stronger effect than plant functional groups on the composition of bacterial communities (Fig. 2). Water/precipitation was also the most relevant factor structuring plant communities in this semi-arid steppe [45], as in general it is the key factor structuring biological communities in arid and semi-arid ecosystems [46]. While pH has been shown to be the primary driver of soil bacterial community patterns across different ecosystems [47], [48] and carbon availability has been reported as the key factor determining the relative abundance of different bacterial taxa [49], the importance of water availability/precipitation in structuring soil microbial communities has not been so widely acknowledged (but see [50]). However the microbial physiological response to water stress and the effect on microbially mediated biogeochemical processes has been comprehensively studied. While dry soils limit substrate diffusion and consequently, microorganisms suffer from resource limitation [51], increasing soil moisture increases the rates of aerobic processes until oxygen limitation [52]. As a matter of fact, the moisture niche of soil microorganisms is highly conserved and it has been suggested that dry-adapted populations tend to be generalists [53].

Overall, soil bacterial diversity and community similarity in this semi-arid ecosystem responded to the variation in water content rather than to differences in plant functional diversity. As we only assessed the bacterial composition in this study, we cannot rule out the possibility that plant functional groups are affecting the activity and function of the belowground microbes. In fact, previous studies have found an association between plants and both microbial functional diversity [54] and nitrogen-cycling genes [21] in steppe grasslands. As the precipitation in this area has been predicted to increase in the future [55], it is crucial to investigate how soil microbial diversity and ecosystem functions will shift in a changing environment.
